# Wisconsin State Medical Society

**Published:** 1872-08-15

**Authors:** 


### WISCONSIN STATE MEDICAL SOCIETY.

The Wisconsin State Medical Society held its regular annual session in the city of Fon du Lac, commencing on the 19th of June and continuing three days, Dr. J. Favill, its President, in the chair.

The attendance was good, and the number of applicants for membership showed a gratifying appreciation of the estimation in which the Society is held by the profession of the State.

An address of welcome was delivered by Dr. E. L. Griffin, which was followed by some necessary routine work.

Reports of Standing Committees were rendered as follows :

On Surgery, several interesting papers were read, and in part illustrated, among which were the following :

A report from Dr. Meacham, embracing—A Pyrogenic Ovarian Cyst, Protracted Use of Chloroform, Resection of Tibia and Fibula in a Case of Viciously United Fracture, and a Case of Senile Gangrene.

From Dr. Lenn, a lengthy and valuable paper on Necrosis and its Treatment.

On Practice, papers were presented as follows :

By Dr. Manley, one touching on the Pathology and Therapeutics of Diabetes, Rheumatism, Cerebro-Spinal Meningitis, Epilepsy, Influenza, and Indigestion.

By Dr. Ferrin, on Small-pox and Vaccination ; and

By Dr. J. C. Meacham, on Encephaloid Cancer.

On New Remedies, an elaborate paper on Hydrate Chloral, by Dr. J. J. Brown, led to a discussion which revealed diversity of opinion and independent thought.

On Diseases of the Eye, Dr. E. W. Bartlett gave a paper on Iritis.

After these came reports of Special Committees, and voluntary papers, among which were the following :

Two papers by D. C. Davies, on Gynæcology, and a successful case of Ovariotomy.

By Dr. C. Linde, on Diseases of the Skin.

By Dr. Waterhouse, an essay on Enervation.

By Dr. I. Nichols, a paper on the Laryngoscope, with exhibition of different varieties of the instrument.

Dr. J. C. Davis, a report of a case on Paracentesis Thoracis in a Child: Recovery.

By Dr. Wigginton, on Hydrate Chloral.

By Dr. Witter, two papers, entitled Urethral Stricture and Urinary Fistula, and Cerebro-Spinal Meningitis.

By Dr. Marston, a history of three cases of Placenta Prævia.

By Dr. Conant, on Cerebro-Spinal Meningitis.

By Dr. Brett, on Compound Fracture, with loss of an inch of the Tibia, and subsequent recovery with use of the limb.

By Dr. Cory, on the Use of Anæsthetics.

By Dr. Brown, two papers on Suggestions on the Use of Anæsthetics in Midwifery, and on Intussusception.

By Dr. Brenton, a case of Post partem Hemorrhage.

By Dr. J. E. Davies, on Correlation of Forces in Physiology and Medicine.

By Dr. Vivian, a case of Fractured Skull with Depression, and its Consequences, himself being the subject.

By Dr. Griffin, a case of Cæsarean Section.

The foregoing papers and reports were prepared with manifest care, were well received, and will appear in full in the published transactions, together with an obituary notice of Dr. Mason C. Darling, the first president of the Society, by Dr. Griffin ; also a biographical sketch of Dr. J. H. Hyde.

At the afternoon session of the second day President Favill gave his annual address, on "The Relation the Profession holds, and ought to hold, toward the Community;" an able and eloquent production.

During the session several resolutions looking to the advancement of medical science were adopted, of which the more important were—one memorializing the Legislature for the appointment of a State Board of Health, in accordance with a movement originating in the American Medical Association, and one in reference to the examination of applicants for the study of medicine, as regards their educational attainments, with a view to raising the standard of scholarship.

A resolution was also adopted authorizing the Secretary to hold copies of the published "Transactions" for sale at $1 per copy.

The election of officers for the ensuing year resulted as follows :

*President*—Dr. H. Van Dusen, of Mineral Point.

*First Vice-President*—Dr. E. L. Griffin, of Fond du Lac.

*Second Vice-President*—Dr. John Dickson, of Allen's Grove.

*Secretary and Treasurer*—Dr. J. T. Reeve, of Appleton.

*Censors*—One year, Dr. D. Mason ; two years, Dr. N. Dalton ; three years, Dr. J. K. Bartlett ; and henceforth one to be elected annually, to hold his office for three years.

The following standing committees were appointed :

*Arrangements*—Drs. J. K. Bartlett, Marks, and Johnson.

*Surgery*—Drs. Dalton, Palmer and Brett.

*Practice*—Drs. Waterbury and Bell.

*Obstetrics*—Drs. Whitney, Marston and Armstrong.

*Pathology*—Drs. E. H. G. Meacham, Brown and A. Clarke.

*New Remedies*—Drs. Lenn, Wm. Fox and Hall.

*Medical Education*—Drs. J. G. Meacham and Mason.

*Diseases of the Eye*—Dr. E. W. Bartlett.

*Ethics*—Drs. Strong, Russell and Marks.

After the appointment of delegates to the American Medical Association, and to the various neighboring State Medical Societies, this Society adjourned to meet in Milwaukee the third Wednesday of June, 1873.

